# Ectopic ACTH Secretion Induced by an Olfactory Neuroblastoma: A Case Report

**DOI:** 10.1155/crie/8834392

**Published:** 2025-07-18

**Authors:** Jérôme Houdu, Roger Jankowski, Duc-Trung Nguyen

**Affiliations:** Department of Otolaryngology and Cervico-Facial Surgery, Centre Hospitalier Régional Universitaire de Nancy, Vandoeuvre-Les-Nancy, France

**Keywords:** Cushing's syndrome, ectopic ACTH secretion, olfactory neuroblastoma, peritumoral cysts, sinonasal tumor

## Abstract

**Background:** Olfactory neuroblastoma (ONB) is a rare tumor of the nasal cavity. It may sometimes present with Cushing's syndrome due to adrenocorticotropic hormone (ACTH) secretion, making it challenging to diagnose.

**Methods:** A 65-year-old man with hypokalemia and general weakness presented to the emergency department for Cushing's syndrome. Brain imaging revealed a tumor originating from the ethmoid bone with peritumoral cysts. The first biopsy suggested an ectopic corticotropic pituitary adenoma or a well-differentiated neuroendocrine tumor. However, the second biopsy confirmed an ONB, as suspected by the otolaryngologist. Treatment consisted of neoadjuvant chemotherapy, surgery, and radiotherapy.

**Results:** The patient was cured of Cushing's syndrome and remained in remission at 10 years of follow-up.

**Conclusion:** An unusual mode of discovering ONB is via the diagnosis of Cushing's syndrome caused by ACTH secretion, which may manifest throughout the course of follow-up. Imaging analysis and discussion with pathologists are essential to achieve an accurate diagnosis.

## 1. Introduction

Olfactory neuroblastoma (ONB) is an uncommon sinonasal tumor that develops from the olfactory epithelium and can extend to the anterior cranial fossa. It represents approximately 5% of nasal cavity cancers, and a recent increase in cases has been reported, which is likely due to better histological recognition [1]. Although its diagnosis is simple when the tumor is well differentiated, it can be challenging when the tumor becomes undifferentiated [2]. Furthermore, it may sometimes present with Cushing's syndrome due to adrenocorticotropic hormone (ACTH) secretion, thus leading to a more challenging diagnosis. The aim of this study was to describe our case concerning ONB and several clues that can lead to the suspicion of ONB rather than other etiologies confronted with a sinonasal malignant process.

## 2. Case Presentation

A 65-year-old man (retired postman) presented to the emergency department for leg edema, weight loss (a weight reduction of 2 kg over a 2-week period), general weakness, and recent development of polyuria and polydipsia. He was administered 60 mg of prednisone once daily for 5 days for a “rhinosinusitis” crisis 2 weeks prior. His medical background was significant for coronary artery bypass graft surgery, high blood pressure treated with lercanidipine (10 mg × 2/day), hyperferritinemia, and chronic rhinosinusitis with nasal polyps (CRwNP), which was diagnosed 2 years prior by an office-based otolaryngologist on nasal obstruction, complete anosmia, some episodes of left epistaxis and complete opacification of the 2/3 superior left nasal cavity and the right maxillary sinus on a sinonasal CT scan. His sinonasal symptoms improved with a short course of general corticosteroid treatment; however, his anosmia was irreversible. He had not previously undergone sinonasal surgery.

During his stay at the emergency department, laboratory results revealed hypokalemia and hyperglycemia. On physical examination, his blood pressure was 100/60 mmHg, his heart rate was 78 beats per minute, and his temperature was 36.9°C. Proximal lower extremity amyotrophy, cutaneous fragility, purpuric lesions on his forearms, and mycosis of the intergluteal cleft were observed. He also presented with hyperirritability and insomnia. The patient was transferred to the endocrinology department for exploration of these disorders. The laboratory findings revealed hypercatabolic hypercortisolism with a marked elevation in plasma cortisol and a loss of the nychthemeral rhythm of the cortisol cycle (Table 1). Dexamethasone suppression tests were negative (i.e., cortisol secretion was not inhibited by dexamethasone administration). A diagnosis of ACTH-dependent Cushing's syndrome was established. In this context, a hypothesis of pituitary adenoma or paraneoplastic syndrome was proposed. The time between the onset of symptoms and the start of cortisol explorations was ~3 weeks. These explorations were therefore not disturbed by the previous intake of cortisol.

**Figure 1:**
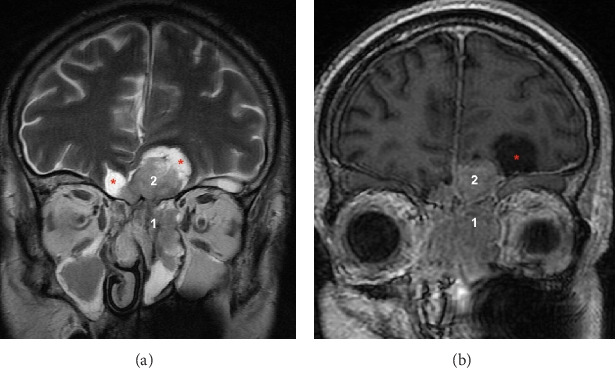
Brain MRI at diagnosis in T2 fast spin-echo (a) and T1 (b) weighted sequences in coronal sections. (1) Intranasal part of the tumor. (2) Intracranial part of the tumor. Red asterisks: peritumoral cysts. This imaging reveals a large, mixed solid and cystic tumor mass, heterogeneous on T2-weighted sequences and isointense on T1, with contrast enhancement. The lesion is both intra- and extracranial, extending across the cribriform plate. Intracranially, it involves the bilateral frontal regions. Extracranially, it invades the nasal cavities. There is evidence of osteolysis of the cribriform plate.

**Figure 2:**
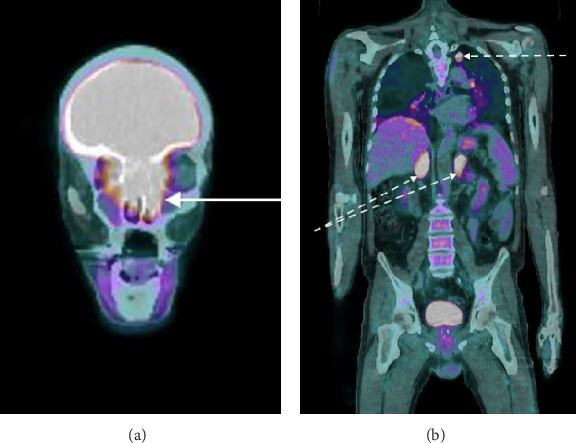
^18^FDG-PET scan at diagnosis showing an intensely hypermetabolic lesion (SUVmax of 8, compared to a hepatic reference of 2) corresponding to a tumoral infiltration originating from the ethmoid, extending into the nasal cavities, without evidence of cervical lymphadenopathy (a, solid white arrow). A nodular left apical pulmonary nodule (SUVmax of 7) is also observed, along with bilateral adrenal hyperplasia (SUVmax of 8) consistent with ACTH-dependent hyperstimulation (b, dotted white arrows).

**Figure 3:**
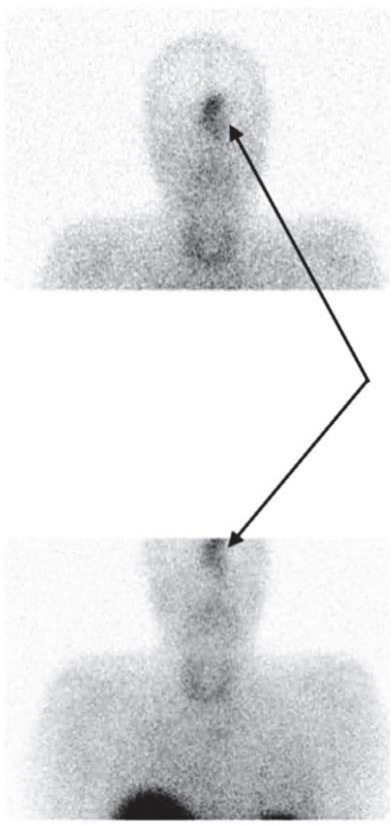
Octreotide scintigraphy revealing increased radiotracer uptake in the ethmoid region and the left nasal cavity (black arrows), with no abnormal uptake observed in the lungs.

A thoraco-abdomino-pelvic CT scan revealed homogenous and symmetric hyperplasia of both adrenal glands without any focal mass. A pituitary MRI revealed a large intra- and extracranial tumor (36 mm × 28 mm × 51 mm). This “dumbbell” mass seemed to have developed from the olfactory cleft. We also observed marginal tumor cysts (Figure 1). The pituitary gland was normal. A whole-body ^18^FDG-PET scan revealed intense hypermetabolism of the nasal mass (SUVmax = 8), a hypermetabolic nodule of the left lung, hypermetabolic bilateral adrenal glands (likely due to hyperstimulation) (Figure 2), and focal hypermetabolism of the L1 vertebra corresponding to a vertebral fracture without retropulsion of the posterior wall, as observed on spine MRI. Octreotide scintigraphy confirmed intense octreotide capture of the nasal tumor, modest octreotide capture of both adrenal glands, and the absence of octreotide capture of the pulmonary nodule (Figure 3). He was treated with metyrapone (1–1.5 g/day) alone then metyrapone + mifepristone (600–1200 mg/day) because of inefficiency of metyrapone. Both drugs were stopped after 5 days because of adrenal insufficiency. Hydrocortisone (20 mg tid) and fludrocortisone (50 µg/day) were subsequently administered. These corticosteroids were stopped 2 weeks later because of difficulty in stabilizing his hypercortisolism, and an urgent bilateral adrenalectomy was performed 1 week later. Histopathological examination confirmed diffuse adrenocortical hyperplasia with no evidence of atypical or malignant cells.

**Figure 4:**
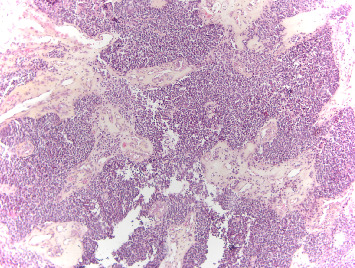
Histological section of the biopsy stained with H&E (original magnification × 10) showing an epithelial lesion organized in sheets with a solid architecture, segmented by a very thin, collagenous, vessel-bearing stroma. The cells are monomorphic, with a high nucleocytoplasmic ratio, round nuclei, finely clumped, and evenly dispersed chromatin and inconspicuous nucleoli.

**Figure 5:**
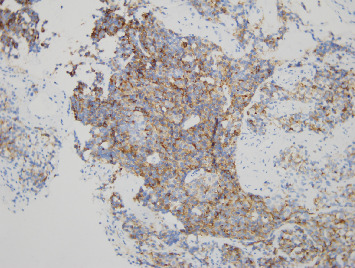
Histological section showing positive ACTH immunostaining of tumoral cells.

The first biopsy of the nasal tumor revealed a lesion consisting of cells in a solid architectural layers, a high nuclear-to-cytoplasmic ratio (Figure 4) with intense expression of neuroendocrine markers, dot-like positivity for cytokeratins, negativity for S-100 protein, and a proliferative index measured using Ki-67 of 2%–3%. The pathologist suggested an ectopic corticotropic pituitary adenoma or a well-differentiated neuroendocrine tumor (such as an atypical ACTH-producing carcinoid). The pathologist reported the limited size of the specimen and the consequent constraints on the reliability of the analysis. Due to the presence of marginal tumor cysts, ONB was suspected by a referral ENT specialist, and a second biopsy was performed. This biopsy revealed a proliferation of cellular nests or a cord organization of monomorphic, small round blue cells with a high nuclear-to-cytoplasmic ratio. The nuclei were slightly anisokaryotic, round or slightly irregular and exhibited a fine chromatin pattern. A small number of Homer–Wright pseudorosettes were present. The tumor cells were intensely and uniformly positive for synaptophysin, chromogranin A, and CD56. Approximately 15% of the tumor cells were positive for the NeuN marker. Immunostaining for S-100 protein was positive, thereby revealing a meshwork of sustentacular cells. Staining for neurofilament, pancytokeratin KL1, and AE1/AE3 markers was negative. Furthermore, immunostaining for ACTH was positive (Figure 5). Pathological features supported the diagnosis of low-grade ONB with ectopic ACTH production.

Due to intracranial invasion of the tumor (T4a stage according to the modified Dulgerov classification; C stage according to the modified Kadish classification), neoadjuvant chemotherapy (three cycles of cisplatin and etoposide) was performed after a multidisciplinary decision was made. However, MRI assessment after chemotherapy revealed slight tumor progression. Endonasal endoscopic tumor removal with anterior skull base resection and reconstruction of osteo-dural defects using a combination of fat and fascia lata was performed. Histological analysis of the surgical specimen confirmed the diagnosis of low-grade ONB (Hyams II), based on a low mitotic index (9 mitoses per 1.75 mm^2^) and the absence of necrosis. Postoperative radiotherapy (66 Gy) was administered. MRI assessment at 3 months revealed no residual signs of the tumor. About 1 month later, the patient was hospitalized for hyperthermia, headache, and acute adrenal insufficiency. Meningitis was diagnosed and treated, despite the absence of identified bacterial agents via lumbar puncture. Brain MRI revealed an intracranial lesion with heterogeneous hyperintensity in T2, hypointensity in T1, peripheral rim enhancement, and perilesional brain edema, thus leading to the diagnosis of an intracranial abscess. He was treated with parenteral antibiotics. Control MRI and CT scans performed at 3 months after this event revealed the disappearance of the intracranial mass. Control visits, including brain MRI and endonasal endoscopic examinations, are annually performed, and the patient is now in remission at 10 years of follow-up. Hormonally, ACTH levels had decreased to 134 pg/mL at 3 months postsurgery and 96 pg/mL at 3 years. Other pituitary functions (TSH, GH, prolactin, LH, FSH) remained within normal ranges during follow-up.

## 3. Discussion

ONB diagnosis is sometimes challenging because this tumor can clinically mimic other benign and malignant tumors based on imaging and histological examinations; moreover, some benign and malignant tumors can also clinically mimic ONB on these same examinations. From a clinical perspective, symptoms of this disease are nonspecific and related to the nasal mass. The most common symptoms include unilateral nasal obstruction, epistaxis, anosmia, and headache [1, 3]. In this patient, the initial diagnosis of CRwNP was erroneous. This diagnosis was based on nasal obstruction, complete anosmia, complete opacification of 2/3 of the superior left nasal cavity on CT scan, and sinonasal symptom improvement under general corticosteroid treatment. However, we observed ethmoidal bone erosion on a sinus CT scan at 2 years before his emergency admission. This observation may have potentially elicited suspicion of a malignant tumor. Despite this, due to the nonspecific symptoms of ONB, the diagnosis of CRwNP was made by a primary ENT specialist without follow-up. The authors point out that the diagnosis of CRSwNP should only be made in the case of bilateral polyps, and that a tumor etiology should systematically be considered in the case of unilateral polyp and lead to an MRI; biopsies will be discussed based on the MRI results [4].

Cushing's syndrome is very rare; however, it can be a manner in which ONB are discovered [5–9] and can erroneously indicate another diagnosis (such as corticotropic pituitary adenoma). The 2021 consensus on diagnosis and management of Cushing's disease reminds that the differential diagnosis between Cushing's disease and Cushing's syndrome by ectopic ACTH secretion relies on a combined approach using ACTH measurement, pituitary imaging, dynamic testing (CRH/desmopressin), inferior petrosal sinus sampling (IPSS), and whole-body imaging (CT, PET), interpreted within the clinical context [10]. Clues that may suggest ectopic Cushing's syndrome include male gender, severe hypokalemia, and very high urinary free cortisol (>5 times the normal) [10]. For many years, bilateral adrenalectomy was considered the first-line treatment for Cushing's syndrome. However, over the past 15 years, the use of steroidogenesis inhibitors has increased, with treatment regimens—either titration or block-and-replace—tailored to the severity of the disease [11].

According to the results of previous studies, the clinical characteristics of ectopic ACTH production by ONB are highly variable and atypical. Ectopic ACTH production by ONB can occur at the time of recurrence of ONB without Cushing's syndrome at the initial diagnosis of ONB [9, 12]. In a nonexhaustive review of the literature conducted by the authors (Supporting Information), just over 20 reported cases described Cushing's syndrome secondary to ONB, occurring across a wide age range (from childhood to 66 years). The syndrome was observed either at initial diagnosis or at the time of tumor recurrence—even in cases where the primary tumor was not ACTH-secreting—often several years after the initial presentation. Physicians should carefully monitor patients with ONB for the development of Cushing's symptoms because the tumor can transform into an ACTH-producing form, even after long-term follow-up [13].

ONB has no specific CT characteristics, and it can initially present as a homogeneous soft tissue mass in the nasal vault that exhibits moderate and uniform enhancement [14]. This scenario can lead to a mistaken diagnosis of CRwNP. On MRI, it is typically hypointense to gray matter on T1-weighted images and intermediate-to-hyperintense on T2-weighted sequences [14]. A typical appearance on MRI is a “dumbbell” mass, with the upper portion located in the intracranial anterior fossa and the lower portion located in the upper nasal cavity [15]. Based on the presence of a sinonasal mass with intracranial extension, marginal tumor cysts highly suggest the diagnosis of ONB in the adult population and have already been described in several studies [14, 16–19]. This clinical scenario can be helpful in guiding the diagnosis of difficult or atypical cases, as occurred in our case. Areas of cystic degeneration may be hyperintense on T2-weighted sequences, as they can obstruct secretions in adjacent sinuses [14]. Due to its location in close proximity to the sella turcica, ONB must be distinguished from pituitary adenomas that have extended downward into the nasal cavity or from intracranial ectopic pituitary adenomas originating from the rest of Rathke's pouch in the sphenoid sinus or clivus bone [20–22].

Histologically, ONB exhibits a lobular architecture composed of primitive neuroblastoma cells with a dense neurofibrillary background and is composed of small round blue cells with hyperchromatic salt-and-pepper nuclear chromatin and small nucleoli [1, 8]. These tumors have a high nucleus-to-cytoplasm ratio and typically exhibit rosettes (Flexner–Winterstein) or pseudorosettes (Homer–Wright) [2]. When the tumor is not well differentiated, the histological differential diagnosis includes sinonasal neuroendocrine carcinoma and sinonasal undifferentiated carcinoma (SNUC), among other diagnoses [2, 23]. Immunocytochemistry is very helpful for diagnosis and usually demonstrates that ONBs are neuron-specific enolase (NSE)-positive in >90% of cases, S-100-positive in approximately 80% of cases, and cytokeratin-negative [1, 2, 24].

## 4. Conclusion

ONB is a rare tumor located at the juncture of the nasal cavity and the base of the skull. An unusual mode of discovering this tumor is via the diagnosis of Cushing's syndrome caused by ACTH secretion, which may manifest throughout the course of follow-up. Imaging analysis and discussion with pathologists are essential to achieve an accurate diagnosis.

## Figures and Tables

**Table 1:** Endocrine data showing hypercortisolism with a marked elevation in plasma cortisol and a loss of the nychthemeral rhythm of the cortisol cycle.

1. Plasma cortisol and ACTH cycle					
Time (clock)	8:00	12:00	16:00	20:00	24:00
Plasma cortisol (µg/L)	896	912	907	886	786
Plasma ACTH (pg/mL)	459	598	611	581	565
2. 24-h urinary-free cortisol level: 15,594 µg (normal: 20–90 µg/24 h)					
3. Dexamethasone suppression test		Minute	Standard	Strong	
Plasma cortisol (µg/L)		1015	1025	714 (cortisol at 8:00)	
4. Hypophysiogram					
Gonadotropic axis: FSH 1 mIU/mL, LH 0.4 mIU/mL (decreased)					
Lactotropic axis: Prolactine T1 10.5 ng/mL, T2 11.2 ng/mL (normal)					
Somatotropic axis: IGF-1 78.2 ng/mL, GH 0.48 mIU/L (normal)					
Thyreotropic axis: TSH 0.15 mIU/L (decreased), free T3 3 pmol/L (decreased), free T4 10.2 pmol/L (normal)					
5. Adrenal androgens					
Δ4-androstendione 19.35 ng/mL (increased)					
SDHEA 545.4 µg/dL (increased)					
17-OH-progesterone 2.78 ng/mL (normal)					
Testosterone 3 ng/mL (normal)					

*Note:* The probable cause of euthyroid sick syndrome is the inhibition of peripheral monodeiodase by corticosteroids.

## Data Availability

All data underlying the results are available as part of the case report and no additional source data are required.

## Supporting Information

Supporting Information Narrative literature review about Cushing's syndrome caused by olfactory neuroblastoma summarizing their features, outcomes, and follow-ups.
