# Supplementary material for: Ectopic ACTH Secretion Induced by an Olfactory Neuroblastoma: A Case Report
Source: Case Rep Endocrinol. 2025 Jul 18;2025:8834392. doi: 10.1155/crie/8834392 (PMC12297143; doi:10.1155/crie/8834392)
Supplement: Supporting Information — Narrative literature review about Cushing's syndrome caused by olfactory neuroblastoma summarizing their features, outcomes, and follow-ups. [file 8834392.f1.docx]

Supplementary material: Narrative literature review about Cushing’s syndrome caused by olfactory neuroblastoma summarizing their features, outcomes and follow-ups.

| Case reports | Age | Sex | History |
| --- | --- | --- | --- |
| Reznik et al.[1]  1987  Belgium | 48 | F | Right nasal obstruction, epistaxis due to a tumor of right nasal cavity. Biopsy confirmed ONB. Surgery was performed 1 month later. 18 months later: recurrence of tumor treated with chemotherapy then surgery 8 months later. Patient refused irradiation. Five years later : Patient presented with delirious state in a clinicobiological feature of Cushing's syndrome. She died about two weeks later. Autopsy found the recurrent ONB secreting ACTH. |
| Arnesen et al.[2]  1994  USA | 36 | F | Delirium and acute psychotic behavior in a context of CS. She presented a nasal "polyp". The resection of this "polyp" which was ONB leading to a resolve of CS. Five years later, she was hospitalized for a florid CS. CT scan demonstrated a large tumor of the right and left nasal cavity which extended through the cribriform plate. Surgery was performed and CS was resolved afterward. |
| Inagaki et al.[3]  2002  Japan | 33 | M | Metastases of retropharyngeal lymph nodes from nasal ONB operated 7 years before. A chemotherapy (cisplatin-etoposide-decadrone) was administered. The patient experienced numbness in his lower extremities, dysgeusia, adynamia, stomatitis, facial and extremity edema, diarrhea, and thrombocytopenia; unexplained hypokalemia was found. It was believed to be adverse effects from chemotherapy. Then he developed psychiatric troubles (anxiety, irritability, insomnia, and psychomotor excitement) and was referred to the psychiatry division. Because of these long-lasting psychiatric manifestations and the hypokalemia, hormonal tests were performed. EAS was diagnosed. The metyrapone was administered and the patient’s Cushing’s syndrome and psychiatric manifestations immediately resolve. |
| Yu et al.[4]  2004  USA | 36 | M | Caucasian man with 5-year history of mild hypertension presented a worsening hypertension, pedal edema, hyperglycemia. Two months later, he was admitted in emergency for CS, supraventricular arrythmia, seizure due to electrolyte abnormalities and prostatitis. Pulmonary imaging showed pulmonary nodules. Biological findings revealed an ACTH-dependent CS. CT scan of the adrenal glands revealed bilateral hyperplasia. No abnormalities on pituitary MRI. ^18^F-FDG PET scan and octreoscan revealed a large area of increased tracer uptake in the ethmoid region and several focal areas in the lungs. Biopsy of pulmonary nodule concluded nocardia nova infection whereas biopsy of ethmoidal tumor confirmed ONB secreting ACTH. He was treated with metyrapone. |
| Kanno et al.^[5]^  2005  Japan | 39 | F | ACTH-dependent CS with pulmonary empyema. CT scan of the chest showed a nodular lesion in the right lung that the biopsy concluded inflammatory fibrotic changes. MRI of the brain failed to identify any pituitary lesions. Repeated MRI of the brain revealed a large tumor in ethmoid sinus involving the intracranial cavity. Surgery was performed. Three years later, EAS increased. A second mass in the left maxillary sinus (who was present in CT scan in September 1998), which was considered as an inflammatory change with chronic sinusitis, increased size. Resection of the lesion of maxillary sinus resolved CS. Histological feature showed ONB secreting ACTH. |
| Fish et al.[6]  2005  USA | 45 | M | East Asian man with 2 months history of epistaxis from a nasal mass, normal pituitary gland. MRI showed a tumor involved cribriform plate. Histopathological feature of biopsy specimen showed atypical cells suggestive of carcinoma. Patient presented a pneumonia and CS. A second biopsy of the nasal mass revealed an ONB staining of the tissue was positive for ACTH. She was treated with ketokenazole and lost of follow up. |
| Butt and Olczak.[7]  2007  UK | 52 | M | Patient was operated for an ONB of the nose extending into the nasopharynx, anterior cranial fossa and right orbit. Five years later, he presented an ACTH-dependent CS. Cerebral MRI confirmed a recurrence of the olfactory neuroblastoma at the left frontal sinus with dural deposits. He was treated with metyrapone 500 mg tds. |
| Josephs et al.[8]  2008  USA | 48 | M | Patient presented to the emergency department for leg edema, blurred vision, and general weakness for 1 week. The progressive hyperpigmentation and facial rounding were occurred over the past 2 years. EAS was suspected after hormonal tests. A pituitary MRI was negative; however, there was a large, aggressive mass centered in the ethmoid and extended through the cribriform plate and pushed against the inferior portion of the frontal lobe. The lesion was neuroendocrine in origin was confirmed by a biopsy specimen. Gross total resection was performed. ACTH-secreting ONB was diagnosed. |
| Koo et al.[9]  2008  Korea | 66 | F | Patient presented EAS after 2 years since diagnosis of ONB, but she had refused curative operation. Pituitary MRI showed no lesion. The nasal tumor had lightly increased in size on head and neck MRI scans. There was no suspected lesion on chest and abdominal CT scan. The hyperplasia of both adrenal glands without any focal mass was observed. Surgery was performed. ACTH-secreting ONB was diagnosed. After surgery, the CS was resolved. |
|  | 37 | F | ACTH-dependent CS with a tumor in the nasal cavity diagnosed as an ONB. The immunostaining for ACTH of the biopsy from her main mass was negative. The ONB was treated by chemotherapy and radiotherapy. The whole body ^18^FDG-PET-scan at 4 months after radiotherapy showed no hypermetabolic lesion. Patient refused surgery after radiotherapy. A sinus MRI 2 years later revealed a decrease in size of the mass. The AES was resolved without any medication. |
| Hodish et al.[10]  2008  USA | 48 | M | Patient with "chronic sinus congestion" treated with nasal polypectomy 2 years earlier presented EAS. CT of the abdomen and pelvis and pituitary MRI were negative. However, there was a mass protruding through the cribriform plate to the right of midline, pushing the inferior aspect of the frontal lobe superiorly. Biopsy results confirmed ACTH-secreting ONB. Surgery was performed followed by adjuvant radiotherapy. CS was resolved. |
| Lin et al.[11]  2009  Taiwan | 64 | F | Asian woman with history of diabetes mellitus, high blood pressure, dyslipidemia. Four months prior to admission, she presented a general weakness, obtunded mental state. Physical examination produced unremarkable findings except ulcerative herpes zoster patches over the right anterior chest wall. Cerebral CT and MRI showed a mass involving the upper clivus and sphenoid sinus. Endocrine tests allowed to diagnose an EAS. |
| Mintzer et al.[12]  2010  USA | 55 | M | 1992: Caucasian man was diagnosed with ONB and had underwent a craniofacial resection; 1996: relapse with metastases of cervical lymph nodes that treated by surgery. Patient refused radiothepay and chemotherapy; 1998: relapse at left submandibular lymph nodes. Surgery followed radiotherapy were performed; 2002: progression in the posterior nasopharynx; 2004: Chemotherapy by etoposide + carboplatin with minor response; 2007: ectopic ACTH syndrome and started on ketoconazole, 400 mg three times a day, along with hydrocortisone replacement; July 2008: intracranial progression. He underwent craniotomy with a partial resection. |
| Galioto et al.[13]  2011  Italy | 3 | M | Diagnosis of ONB at 10 months of age. Surgery was performed. He presented a moon face and a hypercortisolemia but no diagnosis of CS. At 28 months of age: CS with increased ACTH. At 32 months of age: right parapharyngeal lymphadenectomy. Histological feature confirmed lymph nodal ONB metastasis. Twelve months after surgery: normalization of endocrine tests. |
| Han et al.[14]  2012  USA | 59 | M | Nasal tumor associated with hypokalemia, hypertension, and hypergly­cemia. The biopsy showed poorly differentiated carcinoma with neuroendocrine fea­tures. Carboplatin and etoposide chemotherapy was administered. During the second cycle of treatment, CT-scan for abdominal pain showed a perforated diverticulum and bilateral adrenal hyperplasia. He underwent a hemicolectomy with colostomy. A work-up for CS was initiated and confirmed ACTH-dependent CS. No abnormalities on pituitary MRI. Hypokalemia and poor wound healing persisted. CT and ^18^F-FDG PET showed bilateral cervical lymphadenopathy and a mass of right ethmoidal sinus. Urgent bilateral adrenalectomy was performed for a worsening of clinical condition. Surgery for ethmoidal mass was performed 2 months later. Histological analysis concluded ectopic ACTH secretion from ONB. CS was resolved afterward. |
| Rodgers et al.[15]  2012  USA | 51 | M | Patient with unremitting headache, anosmia, nasal congestion for several months. Brain MRI showed a nasal mass extending into anterior cranial base through the cribriform plate. ONB was confirmed and treated by surgery and radiotherapy. Five years later, he was admitted in emergency for right shoulder pain, severe hypokalemia. ACTH levels elevated. Abdominal MRI showed bilateral adrenal hyperplasia. Brain MRI revealed an extradural contrast-enhancing lesion. Craniotomy for resection of the new contrast enhancing extradural lesion. Histopathological feature concluded ONB. The primary resection revealed focal positive staining for ACTH while the secondary specimen was diffusely and strongly positive for ACTH stain. |
| Mayur et al.[16]  2014  USA | 19 | M | Hispanic man with a 4-month history of progressive visual changes (excessive tearing, swelling around the right eye, and decreased vision), right nasal obstruction, headaches, involuntary weight gain, upper body fat distribution changes, and pruritic skin rash. A work-up for CS confirmed ACTH-dependent CS. Imaging showed an ethmoidal mass with orbital and brain extension. Patient received 2 cycles of induction chemotherapy (cisplatin/etoposide) followed by surgical debulking then radiotherapy. Histological analysis concluded ectopic ACTH secretion from ONB. No evidence of recurrence 2,5 years after the diagnosis with normalization of endocrine tests. |
| Kobayashi et al.[17]  2018  Japan | 52 | F | History of ONB treated with surgery and radiation therapy 10 years before then stereotactic radiosurgery and chemotherapy (paclitaxel, carboplatin, and cetuximab) 4 years later for a relapse of the ONB. The patient presented with Cushingoid appearance with progressive lower limb paralysis and general fatigue. One month later, urgently hospitalized due to acute pleuritic chest pain on inspiration. CT revealed left lower lobular consolidations and a contralateral nodule in the right middle lobe. MRI showed that there were no remarkable findings in the pituitary gland, and the ONB had slightly increased in size in the right nasal cavity and the right ethmoid sinus over 3 years. The lungs were suspected as the ACTH source due to high levels of progastrin-releasing peptide and progressive pulmonary consolidation with a contralateral nodule, suggesting small cell lung cancer. Histological examination from bronchoscopy revealed no evidence of malignancy, and *Nocardia cyriacigeorgica* was isolated from bronchoalveolar lavage fluid. Sulfamethoxazole/trimethoprim improved her pulmonary lesions. Somatostatin receptor scintigraphy revealed strong tracer uptake in the ONB lesions without uptake in the lung, indicating that the origin of the EAS was the olfactory tumor. However, histological examination of ONB specimens resected 10 years earlier showed no intracytoplasmic immunopositivity for ACTH. |
| Kadoya et al.[18]  2018  Japan | 50 | M | Abnormal visual field in 2011 due to ONB of sinonasal cavity. Chemoradiotherapy was performed. Tumor had extended to the opposite site of the sinonasal cavity by 2014. By January 2015, hypokalemia, hyperglycemia, and eosinophil and granulocyte decrease and Cushingoid appearance. |
| Yu et al.[19]  2018  UK | 55 | M | One-year history of progressive sore throat, postnasal drip, excessive fatigue, generalized muscle weakness and reduced exercises tolerance. Increase of truncal weight gain, difficulty climbing stairs, culminating in a fall and hospitalization. Clinical examination found Cushing’s feature. Intermittent episodes of confusion. Loss of sense of smell and taste. MRI showed a left-sided nasal mass. Endonasal endoscopic resection confirming ONB and followed by radiotherapy. |
| Decaestecker et al.[20]  2019  Belgium | 41 | F | Cushingoid appearance with symptoms developed over 5 months and included rounding of the face, alopecia of the scalp, hirsutism on the upper lip, easy bruisability, amenorrhea and proximal muscle weakness of the lower limbs. She mentioned a pressing headache and slight deterioration of vision. ONB was found, treated by trans-nasal endoscopic resection following radiotherapy. Hydrocortisone dose was tapered and finally stopped 4 months after surgery. |
| Familiar and Azcutia.[21]  2019  Spain | 31 | M | Two-year history of resistant hypertension requiring 4 antihypertensive drugs. Typical Cushing appearance and rapid weight gain lead to an endocrinologic evaluation. A pituitary MRI revealed a large mass in the skull base. Patient refused surgery and was treated with neoadjuvant chemotherapy (etoposide and cisplatin for three cycles) followed by a full course of fractional radiotherapy. A new MRI revealed mass stability persisting in the last follow-up 1 year after radiotherapy. |
| Chung et al.[22]  2020  Korea | 46 | M | The patient presented with hyposmia and Cushingoid feature. On laboratory test, hypokalemia was identified, and serum cortisol and plasma ACTH levels were in favor of CS. Brain MRI and ^68^Ga-DOTA TEP suggested ONB. He was treated by surgery and radiotherapy. CS disappeared after the surgery. |
| Abe et al.[23]  2021  Japan | 40 | F | Olfactory neuroblastoma surgically removed but recurred six years later, at which point it was again resected with the addition of topical radiation therapy. At 40 years old, the patient developed central obesity, hypokalemia, leukocytosis, and diabetes mellitus. Laboratory tests confirmed the EAS. Octreotide scintigraphy and CT scan showed cervical lymph node metastasis confirmed by biopsy. The patient was treated by metyrapone, hydrocortistone and neck dissection. The EAS decreased after the surgery. |
| Gillette et al.[24]  2022  USA | 23 | F | Patient with known “nasal polyps” and a recent seizure presented with diplopia, proptosis, extraocular motility deficits, and stigmata of CS. CT showed a left sinonasal mass extending into the orbit. Laboratory evaluation revealed refractory hypokalemia and significantly elevated adrenocorticotropic hormone. Histopathologic exam confirmed the diagnosis of ONB. She was treated by neoadjuvant chemotherapy (cisplatine + etoposide) but died from sepsis before surgery. |
| Ozhan et al.[25]  2023  Turkey | 1 | NA | A child presented with nasal obstruction at the age of 10 months. Polypoid mass obstructing the right nasal passage was detected. MRI showed a lesion limited within the nasal cavity. The lesion was completely removed by nasal endoscopic surgery. The pathologic examination revealed a diagnosis of esthesioneuroblastoma. No adjuvant treatment was administered. At 28 months of age, he presented with rapid weight gain. Laboratory data were consistent with Cushing’s syndrome (CS). High-dose dexamethasone suppression test and imaging studies led to think of ectopic ACTH syndrome originated from ENB relapse. After partial resection of the tumor, ketoconazole treatment was started along with chemotherapy. Hypercortisolemia was kept under control with ketoconazole treatment as long as the treatment was maintained. |

*M : male ; F : female ; NA : not available ; ONB : olfactory neuroblastoma ; CS : Cushing’s syndrome ; EAS : ectopic ACTH syndrome*

***References***

[1] Reznkik M, Melon, J, Lambricht, M., et al. Tumeur neuroendocrine de la cavité nasale (esthésioneuroblastome) - A propos d’un cas avec syndrome de Cushing paranéoplasique. *Annales de pathologie* 1987; 7: 137–142.

[2] Arnesen MA, Scheithauer BW, Freeman S. Cushing’s Syndrome Secondary to Olfactory Neuroblastoma. *Ultrastructural Pathology* 1994; 18: 61–68.

[3] Inagaki M, Akizuki N, Kugaya A, et al. Metyrapone for Cushing’s Syndrome. *AJP* 2002; 159: 1246–1246.

[4] Yu J, Koch CA, Patsalides A, et al. Ectopic Cushing’s Syndrome Caused By An Esthesioneuroblastoma. *Endocrine Practice* 2004; 10: 119–124.

[5] Kanno K, Morokuma Y, Tateno T, et al. Olfactory Neuroblastoma Causing Ectopic ACTH Syndrome. *Endocr J* 2005; 52: 675–681.

[6] Fish S, Tapino E. Ectopic ACTH syndrome caused by olfactory neuroblastoma. *Resid staff physician* 2005; 51: 30–33.

[7] Butt MI, Olczak SA. Cushing Syndrome Secondary to Ectopic Adrenocorticotropic Hormone Secretion From Recurrent Olfactory Neuroblastoma: *The Endocrinologist* 2007; 17: 160–161.

[8] Josephs L, Jones L, Marenette L, et al. Cushing’s Syndrome: An Unusual Presentation of Olfactory Neuroblastoma. *Skull Base* 2008; 18: 073–076.

[9] Koo BK, An JH, Jeon KH, et al. Two Cases of Ectopic Adrenocorticotropic Hormone Syndrome with Olfactory Neuroblastoma and Literature Review. *Endocr J* 2008; 55: 469–475.

[10] Hodish I, Giordano TJ, Starkman MN, et al. Location of ectopic adrenocortical hormone‐secreting tumors causing Cushing’s syndrome in the paranasal sinuses. *Head & Neck* 2009; 31: 699–706.

[11] Lin H-H, Lin C-M, Tseng H-K. Refractory Post-Herpetic Neuralgia As An Initial Presentation Of Olfactory Neuroblastoma-Related Ectopic ACTH Syndrome. *International Journal of Gerontology* 2009; 3: 81–84.

[12] Mintzer DM, Zheng S, Nagamine M, et al. Esthesioneuroblastoma (Olfactory Neuroblastoma) with Ectopic ACTH Syndrome: A Multidisciplinary Case Presentation from the Joan Karnell Cancer Center of Pennsylvania Hospital. *The Oncologist* 2010; 15: 51–58.

[13] Galioto S, Di Petrillo A, Pastori M, et al. Metastatic Esthesioneuroblastoma Secreting Adrenocorticotropic Hormone in Pediatric Patients. *The journal of Craniofacial Surgery* 2011; 22: 1924–1929.

[14] Han JY, Mirsadraei L, Yeh MW, et al. Bilateral Adrenalectomy: Lifesaving Procedure in Severe Cushing Syndrome. *Endocrine Practice* 2012; 18: e85–e90.

[15] Rodgers S, Moshel Y, Mikolaenko I, et al. Recurrent Esthesioneuroblastoma Presenting as an ACTH Paraneoplastic Syndrome. *IJNS*; 8. Epub ahead of print 2012. DOI: 10.5580/2b51.

[16] Mayur N, Bordoni RE, Locandro D, et al. Cushing’s Syndrome Due to Ectopic Adrenocorticotrophic Hormone Production by Olfactory Neuroblastoma. *Endocrine Practice* 2014; 20: e47–e52.

[17] Kobayashi K, Asakura T, Ishii M, et al. Pulmonary nocardiosis mimicking small cell lung cancer in ectopic ACTH syndrome associated with transformation of olfactory neuroblastoma: a case report. *BMC Pulm Med* 2018; 18: 142.

[18] Kadoya M, Kurajoh M, Miyoshi A, et al. Ectopic adrenocorticotropic hormone syndrome associated with olfactory neuroblastoma: acquirement of adrenocorticotropic hormone expression during disease course as shown by serial immunohistochemistry examinations. *J Int Med Res* 2018; 46: 4760–4768.

[19] Yu K, Roncaroli F, Kearney T, et al. Ectopic Cushing’s syndrome secondary to olfactory neuroblastoma. *Acta Neurochir* 2018; 160: 1023–1026.

[20] Decaestecker K, Wijtvliet V, Coremans P, et al. Olfactory neuroblastoma (esthesioneuroblastoma) presenting as ectopic ACTH syndrome: always follow your nose. *Endocrinology, Diabetes & Metabolism Case Reports*; 2019. Epub ahead of print 17 October 2019. DOI: 10.1530/EDM-19-0093.

[21] Familiar C, Azcutia A. Adrenocorticotropic Hormone-Dependent Cushing Syndrome Caused by an Olfactory Neuroblastoma. *Clin Med Insights Endocrinol Diabetes* 2019; 12: 117955141982583.

[22] Chung YS, Na M, Ku CR, et al. Adrenocorticotropic Hormone-Secreting Esthesioneuroblastoma with Ectopic Cushing’s Syndrome. *Yonsei Med J* 2020; 61: 257.

[23] Abe H, Suwanai H, Kambara N, et al. A Rare Case of Ectopic Adrenocorticotropic Hormone Syndrome with Recurrent Olfactory Neuroblastoma. *Intern Med* 2021; 60: 105–109.

[24] Gillette WM, Hubbard DC, Waters JN, et al. Esthesioneuroblastoma presenting with orbital signs and ectopic adrenocorticotropic hormone syndrome. *Baylor University Medical Center Proceedings* 2022; 35: 245–247.

[25] Özhan B, Cakar Y, Gülten G, et al. An exceptionally rare case of Cushing’s syndrome caused by ectopic ACTH syndrome due to olfactory neuroblastoma in childhood. *Journal of Pediatric Endocrinology and Metabolism* 2023; 36: 513–516.
